# Diet, Oral Hygiene Habits, and Approach to Dental Visits of Early School-Aged Children during the COVID-19 Pandemic and Possible Long-Term Health Consequences

**DOI:** 10.3390/jcm12175690

**Published:** 2023-08-31

**Authors:** Natalia Torlińska-Walkowiak, Karolina Łukaszewicz, Alicja Morawska, Anna Sowińska, Tamara Pawlaczyk-Kamieńska, Justyna Opydo-Szymaczek

**Affiliations:** 1 Department of Pediatric Dentistry, Poznan University of Medical Sciences, 60-812 Poznan, Poland; 2 Scientific Circle at Department of Pediatric Dentistry, Poznan University of Medical Sciences, 60-812 Poznan, Poland; 3 Department of Computer Science and Statistics, Poznan University of Medical Sciences, 60-806 Poznan, Poland; 4 Department of Risk Group Dentistry, Pediatric Dentistry, Poznan University of Medical Sciences, 60-812 Poznan, Poland

**Keywords:** diet, hygienic habits, children, dental visits, COVID-19 pandemic, caries

## Abstract

Introduction: Early school-aged children are in a transitional phase from primary to permanent dentition. Established dietary and oral hygiene habits will influence the condition of the oral cavity in the future. Aim: This study aimed to evaluate alterations in early school children’s dietary and oral hygiene practices during the COVID-19 pandemic and to anticipate potential long-term health implications. Material and Methods: This cross-sectional online study involved guardians of Polish children aged 6–10 years, living in Western Poland, who were socially isolated at home during the COVID-19 pandemic. A total of 180 guardians were invited to participate in this study. The questionnaire included 17 questions divided into four different sections—the child’s anthropometric data, dietary habits, oral health, and attitude to dental visits. Results: The survey was completed by 106 guardians. The mean (standard deviation) age of the children was 8.12 (0.93) years (range 6–10 years). Overall, 24.5% of the surveyed individuals reported buying healthy products (fresh vegetables, fruit) more frequently during the pandemic. Furthermore, 35.8% admitted to snacking between meals more frequently and 16.0% less frequently. Almost one-quarter of the parents acknowledged that their children were less motivated to maintain oral hygiene during the pandemic and 28.3% declared a lower frequency of visits to the dentist from the outbreak of the pandemic for two main reasons: a fear of coronavirus transmission and economic reasons. Conclusions: In the group studied, the results of the assessment indicate that the COVID-19 pandemic had some effects on oral health that may lead to an increased risk of oral disease development, such as tooth decay in children. None of the respondents noticed an increase in their child’s motivation about good oral hygiene despite spending more time at home. The irregularity of follow-up visits for one-third of the respondents hindered preventive measures and the continuation of dental treatment.

## 1. Introduction

In response to the global COVID-19 pandemic, Poland, like many countries, implemented a series of restrictive measures to curb the spread of the virus and safeguard public health. These measures had a notable impact on various sectors, including oral health services.

Government-imposed restrictions, including social distancing, lockdowns, and quarantine protocols, were crucial to reducing virus transmission and treating those infected [1]. However, these measures had unintended consequences on the accessibility and utilization of dental services, resulting in a complex set of challenges for both oral health professionals and the general population [2].

One of the primary consequences of the pandemic was a noticeable reduction in the number of patients seeking scheduled dental procedures and preventive visits. The imposition of social distancing and quarantine measures led to hesitancy among patients to attend healthcare facilities, including dental clinics, unless faced with urgent dental issues. Many individuals postponed routine check-ups, cleanings, and elective procedures due to concerns about potential exposure to the virus. At the same time, it has been shown that oral health affected the course of the COVID-19 disease. A more severe course of the disease was documented in patients with periodontal problems and poor oral hygiene [3]. As the situation improved and vaccination efforts progressed, Poland began to gradually lift restrictions. By 16 May 2022, many of the pandemic-related restrictions affecting dental services were eased, allowing dental practices to resume normal operations [4].

The role of the family in children’s oral health and the parents’ impact on the prevention of early childhood caries are well established [5,6]. Parents play a vital role in filtering the interaction between children and their environment with feeding habits, oral hygiene care, and other preventive practices [7].

The oral health of children up to 12 years old is an important issue, and reducing levels of decay in this group would have a positive impact on their overall health [8]. Early school-aged children are in a transitional phase from primary to permanent dentition. This is also the time for changes in oral hygiene and dietary practices—with unsupervised toothbrushing and increased consumption of cariogenic foods and beverages while at school or home, in turn causing a higher risk for developing caries. Therefore, special attention should be given to school-aged children regarding their oral hygiene and dietary practices [9].

The long period of school closures in Poland, with remote lessons from home and changes in daily routine, may have caused some children to become more susceptible to dental caries risk behaviors, such as frequent snacking, a diet rich in sugars, and irregular tooth brushing. On the other hand, the threat of contracting a viral disease may have influenced the previous habit of regular visits to dental surgery.

The COVID-19 pandemic caused us to realize that such a global epidemic can lead to a disruption in the functioning of a community. Therefore, knowledge gained concerning the dental behavior of patients can help improve health management. Hence, this study aimed to evaluate alterations in early school children’s dietary and oral hygiene practices and dental visits before and during the COVID-19 pandemic and to anticipate potential long-term health implications. We hypothesized that parents were more willing to take a child to a dental office for a check-up, treatment of caries, and emergency treatment before the pandemic.

## 2. Materials and Methods

This cross-sectional study involved guardians of Polish children aged 6–10 years who were socially isolated at home during the COVID-19 pandemic. The subjects were invited to participate in this study by answering a questionnaire available on the Google Forms platform from 3 February to 17 March 2021. A link to the questionnaire was sent to four randomly selected primary public schools in the Greater Poland Province with a request for distribution among parents of 180 students of early school education age. The selection of schools was performed using a random sampling technique with the use of Statistica (version 13.1) software based on the school listings provided by the Ministry of Education and Science. Four target schools were chosen to ensure appropriate age representation in the sample. The inclusion criteria were: (1) born between 2011 and 2016 and (2) residency in the Greater Poland Province. We chose children younger than eleven years of age because up to the age of 8–10, it is recommended that guardians help with oral hygiene [6,10]. We intentionally chose not to exclude children with general health issues from our research, so our questionnaire did not include specific inquiries about general health issues. Our decision was based on the premise that pandemic-related changes in diet and oral hygiene habits could be universally experienced, regardless of specific health conditions.

The sample size calculation was performed with G*Power 3.1.9.2 software. We expected a large effect size (i.e., an odds ratio of change from one answer in the questionnaire to another that equaled 3 or 0.333) and a proportion of discordant pairs of 30%. To achieve a power of 0.80 with α set at 0.05, a minimum of 100 subjects were required to detect the expected difference with the use of McNemar’s test.

The anonymous online survey, based on a previous survey [11], was developed by the authors to consider how guardians perceived their dietary habits, their children’s oral hygiene, and their attitudes toward dental visits before the pandemic compared to the period during social distancing. The prefinal version of the questionnaire was pilot-tested on 10% of the calculated sample, with the aim of checking aspects related to the level of understanding and coherence of the questions. Finally, after some small necessary adjustments, the final form of the questionnaire was created. Considering that all questions were formulated by the researchers for this particular project, and due to the recent emergence of the COVID-19 pandemic, the survey had not undergone prior validation.

The questionnaire included 17 questions divided into four different sections: (1) the child’s anthropometric data (5 questions); (2) the child’s dietary-habit information before and during the COVID-19 pandemic (4 questions); (3) oral health before and during the COVID-19 pandemic (3 questions); and (4) the attitude toward dental visits in connection with the pandemic (5 questions). The demographic data included the age and gender of the child, location (urban or rural), the guardian’s level of education (primary, secondary, higher), and the level of interest in health issues. The translated questionnaire is shown in the Supplementary Materials. The questionnaire was preceded by a consent statement, in which the purpose of the study was specified, and the respondents were informed about the processing, the scientific use of data, and the anonymous nature of the form.

Confirmation was obtained from the Chairman of the Bioethics Committee of the Poznan University of Medical Sciences that the survey described above was not a medical experiment and does not require an opinion from the Bioethics Committee.

Data analysis included the description of the relative and absolute frequencies of the variables. Association tests were performed using Fisher’s exact and Kruskal–Wallis tests for ordinal variables. The data were elaborated with Statistica v. 13.1.

## 3. Results

The survey was completed by 106 guardians; the response rate was 58.9%. Most of the respondents had higher education. Overall, 70% described themselves as being interested in health but without medical education. A demographic breakdown of the studied population is presented in Table 1.

The mean (standard deviation) age of the children was 8.12 (0.93) years (range 6–10 years). Of the total number of children, 59 (56%) were girls, and 47 (44%) were boys. Most of the surveyed individuals inhabited urban areas in the suburbs of a large city.

### 3.1. Influence of the Pandemic and Social Distancing (Suspension of Classroom Activities) on the Eating Habits of the Child and Parents

**Figure 1:**
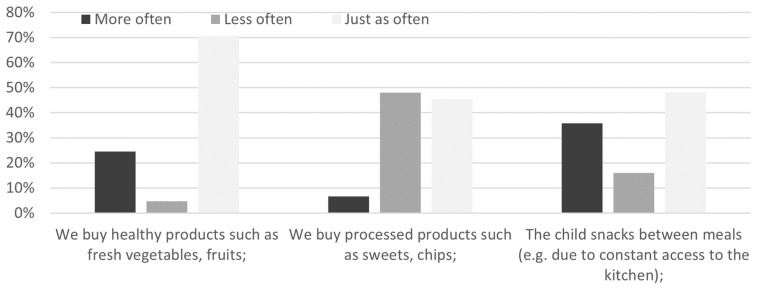
Changes in the quantity and quality of meals eaten during the pandemic.

In the section on meals eaten (Figure 1), 24.5% of surveyed individuals reported buying healthy products (fresh vegetables, fruits) more frequently during the pandemic, and 4.7% reported buying healthy products less frequently. More frequent purchasing of processed snacks was reported by 6.6% of the respondents, while 48.1% believed that they reached for such products less often in the store. On the other hand, 35.8% admitted to snacking between meals more frequently, and 16.0% reported less frequent snacking between meals.

**Figure 2:**
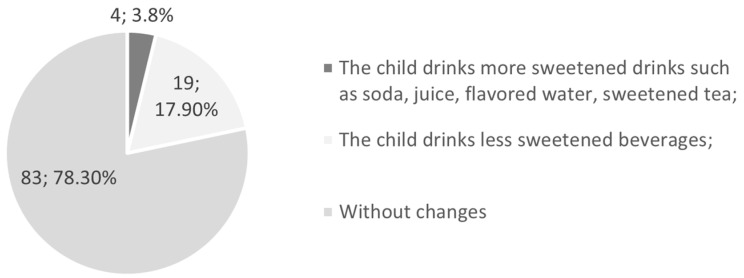
Changes in children’s drinking habits during the pandemic.

Nearly 78% of the respondents did not notice a change in the consumption of beverages by children during the day, and according to the guardians, 17.9% of children drank less sweetened beverages (Figure 2).

**Figure 3:**
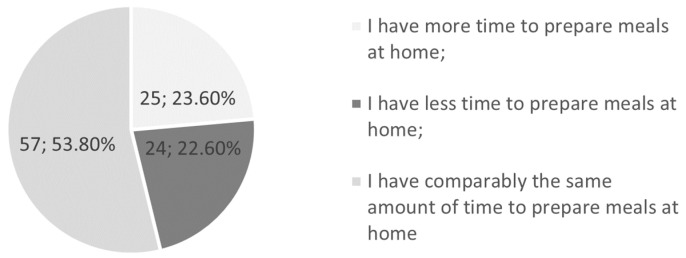
Frequency of meal preparation at home during the pandemic.

The present study also examined the data on preparing meals at home (Figure 3). Overall, 23.6% answered that they had more time to prepare home-cooked meals; a different opinion was shared by a similar group totaling 22.6% of those examined.

The majority of people surveyed (63.2%) did not change their habits regarding ordering ready-made food; 4.7% ordered more often, while 32.1% reported ordering highly processed food (fast food) less often. Thus, the most significant change was declared concerning the decrease in the consumption of processed food.

### 3.2. Influence of the Suspension of Stationary Classes on the Maintenance of Child Oral Hygiene

**Figure 4:**
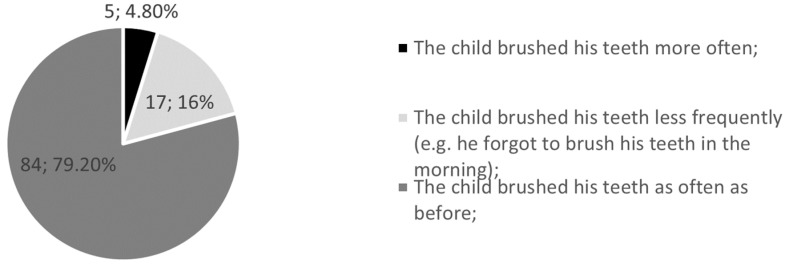
Tooth brushing during the pandemic.

In our survey, guardians were asked if their children’s hygiene behavior changed due to remote learning. The observations show that 16% of children brushed their teeth less often than usual and only 4.8% brushed more often than usual (Figure 4). In addition, almost one-quarter of caregivers (24.5%) acknowledged that their children were less motivated to maintain oral hygiene during the pandemic, while 75.5% were just as motivated as before.

A relatively small percentage of parents (15.1%) changed their habit of supervising the correctness of brushing their children’s teeth, reporting more or less frequent supervision. Almost half of the examined group (47.2%) supervised oral hygiene as often as before. A relatively large subgroup (37.7%) of respondents reported that children always brushed their teeth on their own, without parental supervision.

### 3.3. Attitude toward Visiting the Dentist during the Pandemic

**Figure 5:**
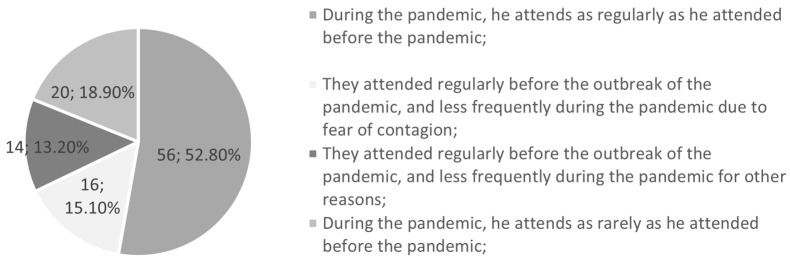
Dental visits before and during the pandemic.

In the third part of the survey, guardians were asked about their attitude to dental visits during the pandemic (Figure 5). Overall, 71.7% said they had not changed the frequency of dental check-ups for their children: they visited the surgery equally regularly or equally rarely at 52.8% and 18.9%, respectively. Most guardians (52.8%) believed that their visits were similarly frequent, while 28.3% declared a lower frequency of visits to the dentist since the outbreak of the pandemic for two main reasons: fear of coronavirus transmission—chosen by 15.1% of respondents—and for other reasons, e.g., economic, selected by 13.2% of respondents.

This study showed differences in the reasons for visiting a dental practice before and during the pandemic.

Respondents admitted to visiting a dental office for a check-up visit more often before the pandemic (*p* = 0.001, McNemar’s test) (Table 2). Still, in an emergency situation due to toothache or dental trauma, parents were willing to arrange a dental visit sooner in the pandemic (*p* = 0.001, McNemar’s test).

Finally, the perceived risk of coronavirus infection in a dental office was examined. Overall, 66% of the respondents believed that the risk of coronavirus infection in a dental office was comparable to the risk in other public places, and 24.7% believed that it was lower. Only 6.6% said that it was significantly higher.

## 4. Discussion

The impact of the pandemic on behavioral changes has become a subject of research interest in recent years [11,12,13,14]. During holidays or days off from school, individuals tend to take a break from their routine and often indulge in different dietary and hygiene habits [10,15]. The lockdown and remote learning period during the pandemic can be likened to a holiday period in terms of changes in routine and lifestyle. However, it is important to note that the pandemic period was also characterized by stress and uncertainty, which were significant factors influencing the behavior of both adults and children. Unlike traditional holiday periods that are typically associated with relaxation and leisure, the pandemic-induced restrictions brought about unique challenges and concerns that affected individual well-being [11,13,14]. Recently published studies indicated that diverse patterns of lifestyle modifications emerged among individuals worldwide during the pandemic [14]. In children, the effects of the pandemic included, among others, inadequate nutrition with a risk of being both overweight or underweight, an addiction to screens, a lack of schooling, and psychosocial difficulties [14].

Regarding changes in eating habits, two primary factors were at play: the confinement at home with the stockpiling of food due to grocery shopping limitations [16] and the constant exposure to COVID-19 news through media sources, which could have induced stress with individuals turning to excessive eating, particularly seeking out sugary “comfort foods” [12,16,17]. These foods, primarily composed of simple carbohydrates, could have helped in alleviating stress by promoting serotonin production [18]. At the same time, the increased amount of time spent at home may have yielded more favorable results, such as a higher frequency of cooking meals instead of relying on fast food options [19].

The cross-sectional online survey conducted by Sidor and Rzymski revealed that Polish citizens instructed to adhere to stay-at-home guidelines during the outbreak-related lockdown exhibited notable changes in their eating behaviors, often displaying a tendency to consume increased amounts of food. Over 43.0% of the adult respondents reported an increase in food consumption, while approximately 52% indicated a higher frequency of snacking. Authors emphasize that these adverse changes may have enduring impacts on human health [13].

An Italian survey carried out on a population of 220 children between 4 to 14 years of age revealed that during the COVID-19 lockdown, there was an increase in caries risk factors compared with protective factors. In the online survey, parents reported frequent consumption of dietary sugars and snacks between meals, incorrect oral hygiene habits, and inadequate topical fluoride prophylaxis [12].

As expected, our study also confirmed significant changes in the adoption of healthy activities related to diet and oral health habits during the pandemic. We observed two opposite patterns: both pro-healthy and unhealthy, marked to varying degrees depending on the field studied. During the pandemic, a quarter of the respondents paid more attention to the content of fruit and vegetables in their diet. At the same time, almost half bought sweets and crisps less often, although every third child snacked between meals more often.

Two different trends regarding changes in pro-health behaviors were also observed in the Polish study by Górnicka et al., in which 36.6% of the respondents showed a decreased intake of fast food, and 48% showed an increased consumption of homemade meals [20]. Although that study was conducted on a group of adults, mainly women up to 40 years of age, the data on fast food consumption are similar to ours. In our survey, almost a third of respondents (32.1%) declared that they had reduced the amount of fast food they consumed during the pandemic, while only 23.6% stated they had more time for preparing homemade meals. What is more, Górnicka et al. found that approximately 19% of respondents increased their consumption of unhealthy food [20]. On the contrary, less than 10% of our respondents admitted to buying sweets and crisps in this study. One study on a pediatric population, conducted by Campagnaro et al., revealed that among those who claimed changes in eating habits, only 33.1% said they were choosing healthier foods, while in our study it was 24.5% [11].

According to the study by Gotler et al. [21], 60.4% of 6- to 10-year-olds admitted to having more snacks per day during the pandemic, of which 19.4% admitted to having more than three additional meals, while in our study, 35.8% of respondents reported snacking between meals more frequently, and 16.0% less often than before the pandemic. In Gotler et al.’s study, 18.9% of participants drank sugary/carbonated/juices/soda more frequently between meals during the COVID-19 lockdown, while in our research, only 3.8% confirmed such an adverse change in eating habits [21]. Restrictions on purchasing snacks and sugary drinks in our survey may be due to limited shopping funds because of reductions in family income after a year of the pandemic, but we did not investigate this particular aspect.

It seems that dietary education and counseling for the guardians should include the cariogenicity of certain foods and beverages, the role of consumption frequency of these substances, and the demineralization/remineralization process.

Regarding child oral hygiene, in Gotler et al.’s. study, 29.3% brushed their teeth fewer times a day [21], while in our research, 16% of children declared brushing their teeth less frequently. It was proven that the benefit of twice daily toothbrushing on caries development in newly erupted first permanent molar teeth is around 50% compared with brushing once a day or less [22]. Thus, more attention to the educational aspect should be exerted. A cross-sectional survey of parents of children aged 3–15 years from Brazil and Portugal showed that child oral hygiene habits changed in 43% cases during the social distancing period of the pandemic. Parents considered oral hygiene as poorer in 22.9% of cases and better in 19.8% of cases [2].

Regarding dental visits, in the study by Campagnaro et al. [11], about 56% of respondents said they were not leaving home to attend medical or dental appointments, and 86% of those who reported that their children experienced dental trauma during the pandemic did not seek dental care. In addition, 24.4% of the children had their dental treatment temporarily suspended [11]. In our study, 28.3% of respondents canceled regular visits. Still, in an emergency due to toothache or dental trauma, parents were willing to arrange a dental visit sooner in the pandemic than before (*p* = 0.001). Nevertheless, only one-third of Polish 7-year-olds attended regular check-ups the year before the pandemic. Reducing this already insufficient number of visits due to the pandemic may lead to a further deterioration in child oral health [23]. Our study results partially support the initial hypothesis. Parents were more willing to take a child for a dental check-up before the pandemic. However, in an emergency situation, such as a toothache or dental trauma, they were more willing to arrange a dental visit during the pandemic.

The results of some studies also show positive trends in people’s behavior during the pandemic. Health awareness among young school children can increase during a period of social isolation, as evidenced by the study by Kołota et al. [24]. Polish primary school children aged 10–16 years were examined at the beginning of the pandemic in June 2020. Within the area of food consumption habits, the number of students who declared ensuring that they ate at least one serving of fruit a day, or at least three servings of fruit most days, or at least one serving of vegetables or salad a day, or often choosing a fruit when they had a snack between meals, actually increased (*p* < 0.05). Participants of this study presented different eating habits than before, and most of the changes were positive [24].

The oral health of humanity has been influenced by various factors throughout history, including diet [25,26], oral hygiene practices [27], changes in environmental conditions [28], and crisis situations such as wars, periods of famine, and pandemics [29]. These factors can have direct or indirect impacts on oral health, such as changes in dietary patterns affecting the prevalence of dental caries or disruptions in access to dental care during times of conflict or crisis [28,29]. Regarding the COVID-19 pandemic, it has undoubtedly had a lasting impact on the health of people globally, including oral health.

Thus, it is important to monitor the long-term effects of the pandemic on establishing new oral hygiene and dietary habits as well as oral health. Healthcare professionals and educators should continue to stress the significance of maintaining good oral health during challenging times like a pandemic. This includes highlighting the link between oral health and overall well-being, encouraging individuals to maintain regular oral hygiene routines, emphasizing the importance of reducing sugary food and beverage consumption, and reminding them that these practices play a critical role in preventing oral health problems. Dental health education should be integrated into school curricula, which will help children understand the importance of oral health from a young age. Parents should be involved in discussions about establishing oral health routines at home, which can help create a consistent and supportive environment for children’s oral health education.

Our research also opens the door to further research inquiries. Exploring the mechanisms behind the changes, such as the influence of stress, disrupted routines, increased screen time, and parental control, offers a promising avenue for future investigation. Understanding the underlying drivers of these trends could contribute significantly to targeted interventions and strategies aimed at promoting positive oral health behaviors among children.

There are several limitations to consider in this study. Firstly, the population size was small, restricted to one province, which could lead to results that may not be representative of the entire population of children in Poland at that age. An additional constraint worth considering is the response rate of the questionnaire, which might impact the ability to generalize our findings. Finally, this anonymous online survey was not validated, although questions contained within it were created to address issues that were important to oral health care policymakers. 

Nevertheless, the novelty of our findings and the role that they could play in the development of oral health education programs should balance these limitations. 

## 5. Conclusions

Our paper presents a unique exploration of the impact of the pandemic on the oral health habits of children in Poland, focusing specifically on the age group of 6 to 10 years old, the age at which there is the greatest increase in caries. Using an innovative questionnaire study conducted among parents, this research sheds light on the multifaceted effects of the pandemic on children’s oral health practices and underscores the complexity of these changes.

In the group studied, the results of this study indicate that the COVID-19 pandemic had some effects on oral health that may lead to an increased risk of oral disease development, such as tooth decay in children.

None of the respondents noticed an increase in their child’s motivation to care for good oral hygiene despite spending more time at home.

The irregularity of follow-up visits for one-third of the respondents hinders preventive measures and the continuation of dental treatment.

## Figures and Tables

**Table 1:** Demographic data of surveyed participants (*n* = 106).

		*n*	%
Participants		106	100
Gender of the child	Male	47	44
	Female	59	56
Characteristics of profession, interests	Medical	14	13
	Medical-related education	12	11
	Not related to the medical profession, but I am interested in health	74	70
	Not related to the medical profession, and I have no interest in health	6	6
Age of the child	10 y.	6	6
	6 y.	2	2
	7 y.	28	26
	8 y.	37	35
	9 y.	33	31
Place of living	Urban	57	54
	Rural	49	46
Education *	Primary	6	6
	Secondary	15	14
	Higher	85	80

* The education system in Poland includes 8-year primary school (primary education); general or technical secondary school, sectoral vocational school, or post-secondary school (secondary education); and Bachelor’s or Master’s degree programs (higher education).

**Table 2:** Association between the guardians’ willingness to take their children to dental appointments before and during the pandemic in relation to dental problems *n*/%.

	In What Situation(s) or for What Purpose(s) Were You Willing to Go to the Dentist with Your Child before the Outbreak of the Pandemic? *n*/%	In What Situation(s) or for What Purpose(s) Are You Willing to Go with Your Child to the Dentist during the Pandemic? *n*/%	*p*-Value *
Toothache	78/74%	97/92%	0.00009
Tooth trauma	80/75%	96/91%	0.0002
Caries	84/79%	92/87%	0.999
Control visit	95/90%	78/74%	0.001

* McNemar’s test.

## Data Availability

The data presented in this study are available upon request from the corresponding author.

## Supplementary Materials

The following supporting information can be downloaded at: https://www.mdpi.com/article/10.3390/jcm12175690/s1, Table S1: Questionnaire sent to caregivers of children aged 6–10 years old.
