# Supplementary material for: Diet, Oral Hygiene Habits, and Approach to Dental Visits of Early School-Aged Children during the COVID-19 Pandemic and Possible Long-Term Health Consequences
Source: J Clin Med. 2023 Aug 31;12(17):5690. doi: 10.3390/jcm12175690 (PMC10488684; doi:10.3390/jcm12175690)
Supplement: Supplementary file 1 [file jcm-12-05690-s001.zip › jcm-2536896-supplementary.pdf]

Table S1. Questionnaire sent to caregivers of children aged 6–10 years old.

Question		
How old is your child?		
Gender of the child	Male; Female	
What is your level of education?		
Characteristics of your profession, interests	Medical;<br>Medical related education;<br>Not related to the medical profession, but I am interested in health;<br>Not related to the medical profession and I have no interest in health	
Where do you live?	Urban, rural areas	
The impact of the pandemic and the suspension of stationary classes on the eating habits of the child and parents		
How has the quantity and quality of meals eaten changed during the pandemic?	We buy healthy products such as fresh vegetables, fruit;<br>We buy processed products such as sweets, crips;<br>The child snacks between meals (e.g. due to constant access to the kitchen)	More often;<br>Less often;<br>Just as often
What changes have you noticed in your child's drinking habits?	The child drinks more sweetened drinks such as soda, juice, flavored water, sweetened tea;<br>The child drinks less sweetened beverages;<br>Without changes	
Due to the pandemic, do you have more time to prepare meals at home (e.g. due to the transition to remote work)?	I have more time to prepare meals at home;<br>I have less time to prepare meals at home;<br>I have comparably the same amount of time to prepare meals at home	
How has the amount of fast food eaten (e.g. ordered for delivery) changed?	Since the outbreak of the pandemic, we have eaten less ready-made, processed food-fast food;<br>Since the outbreak of the pandemic, we have eaten more ready-made, processed food-fast food;<br>Without changes.	
The impact of the suspension of stationary classes on the maintenance of oral hygiene in a child		
Did the child brush his teeth more/less during distance learning?	The child brushed his teeth more often;<br>The child brushed his teeth less frequently (e.g. he forgot to brush his teeth in the morning);<br>The child brushed his teeth as often as before.	
Did your child brush his/her teeth more/less under your supervision?	We more often supervised the correctness and length of tooth brushing by the child during remote learning;<br>Less often we supervised the correctness and duration of tooth brushing by children during distance learning;<br>We supervised the correctness and duration of tooth brushing by the child as often during remote teaching as before the pandemic;<br>The child always brushed his teeth by himself.	
Have you noticed a change in your child's attitude to maintaining oral hygiene when they spent more time at home?	The child had less motivation to maintain hygiene;<br>The child was more motivated to maintain hygiene;<br>The child was just as motivated as before the pandemic.	
Dental visits before and during the pandemic		
Did your child regularly attend dental check-ups before and during the pandemic?	During the pandemic, he has attended as regularly as he attended before the pandemic;<br>They attended regularly before the outbreak of the pandemic, and less frequently during the pandemic due to fear of contagion;<br>They attended regularly before the outbreak of the pandemic, and less frequently during the pandemic for other reasons;	

	During the pandemic, he has attended as rarely as he attended before the pandemic.	
Have you attended your dental check-ups during the pandemic?	Yes;<br>No	
In what situation(s) or for what purpose were you willing to go to the dentist with your child before the outbreak of the pandemic?	Toothache;<br>Tooth trauma;<br>Caries;<br>Control visit.	Yes;<br>No
In what situation/s or for what purpose are have you been willing to go with your child to the dentist during the pandemic?	Toothache;<br>Tooth trauma;<br>Caries;<br>Control visit.	Yes;<br>No
